# Individual Cytokines Modulate the Neurological Symptoms of *ATM* Deficiency in a Region Specific Manner^1^,^2^,^3^

**DOI:** 10.1523/ENEURO.0032-15.2015

**Published:** 2015-08-18

**Authors:** Chin Wai Hui, Karl Herrup

**Affiliations:** Division of Life Science, The Hong Kong University of Science and Technology, Clear Water Bay, Kowloon, Hong Kong

**Keywords:** ataxia-telangiectasia, cell cycle, HDAC4, IL1beta, Purkinje cell, TNFalpha

## Abstract

Ataxia-telangiectasia (A-T) is a multisystemic neurodegenerative disease of childhood caused by the absence of functional ATM (A-T mutated) protein. The cerebellar cortex has the most obvious neuropathology, yet cells in other brain regions are also abnormal. A-T mouse models have been produced that replicate much, though not all, of the complex A-T phenotype. Nongenetic factors, including modulations of the immune status of the animal, have also recently been found to play a role in the disease phenotype. Here we report that these modulations show both cytokine and brain region specificity. The CNS changes induced by broad-spectrum immune challenges, such as lipopolysaccharide (LPS) injections are a complex mixture of neuroprotective (TNFα) and neurodegenerative (IL1β) cytokine responses that change over time. For example, LPS first induces a protective response in A-T neurons through activation of tissue repair genes through infiltration of monocytes with M2 phenotype, followed over time by a set of more degenerative responses. Additional phenotypic complexity arises because the neuronal response to an immune challenge is regionally variable; cerebellum and cortex differ in important ways in their patterns of cellular and biochemical changes. Tracking these changes reveals an important though not exclusive role for the MAP kinase pathway. Our findings suggest brain responses to cytokine challenges are temporally and regionally specific and that both features are altered by the absence of ATM. This implies that management of the immune status of A-T patients might have significant clinical benefit.

## Significance Statement

Ataxia-telangiectasia (A-T) causes early cerebellar neurodegeneration, leading to early loss of motor ability in patients. We show that inflammation specifically accelerates A-T neurological symptoms and this effect is modulated by a subset of proinflammatory cytokine. Long-term recovery studies demonstrate A-T animals fail to fully repair the inflammation induced damage. These findings highlight the gene/environment interactions that drive the final set of A-T symptoms and suggest the potential for immunomodulation as a strategy to treat neurodegenerative diseases.

## Introduction

Ataxia-telangiectasia (A-T) is a multisystemic autosomal recessive disorder associated with progressive neuronal degeneration, cancer susceptibility, hypersensitivity to ionizing radiation, immune deficiency, and sterility (Lavin and Shiloh 1997; Chun and Gatti 2004; Biton et al., 2008). A-T is caused by mutation in the *ATM* (ataxia-telangiectasia mutated) gene, which encodes a 370 kD protein kinase (ATM) that is involved in many cellular behaviors: the DNA damage response, cell-cycle regulation, and cell survival (Biton et al., 2008; Herrup et al., 2013; Li et al., 2013). In the adult CNS, ATM protects neurons by suppressing the cell cycle, repairing damaged DNA (Dar et al., 2006), controlling vesicle trafficking (Li et al., 2009), and maintaining the histone code (Li et al., 2012, 2013). Some, though not all of the neuropathological phenotypes associated with human A-T have been described in *Atm^−/−^* mouse models (Becker-Catania et al., 2000; Gatti et al., 2001; Dar et al., 2006; Li et al., 2011).

The continued expression of proinflammatory cytokines is believed to contribute to the progression of a number of neurodegenerative diseases (Akiyama et al., 2000; Reale et al., 2009; Cappellano et al., 2013). These agents are released in part due to engagement of Toll-like receptors (TLRs) that are expressed in varying patterns on both glial cells and neurons (Bsibsi et al., 2002; Hanamsagar et al., 2012; Amor et al., 2010). Activation of TLRs causes the release of inflammatory cytokines that change the brain microenvironment. During Alzheimer’s disease (AD), elevation of interleukin 1 beta (IL1β) stimulates the expression and processing of the amyloid precursor protein and accelerates amyloid plaque deposition (Forloni et al., 1992; Akiyama et al., 2000). IL1β also induces S100β expression, which triggers neuronal calcium influx, the glial release of other proinflammatory cytokines and increased β-amyloid (Li et al., 2000; Liu et al., 2005). Similar to IL1β, tumor necrosis factor (TNF) levels are correlated with the severity of AD (Paganelli et al., 2002). In human, the levels of circulating and residual TNFα are correlated with cognitive decline (Holmes et al., 2009; Cunningham 2013) and accumulation of amyloid plaques (Montgomery and Bowers, 2012). In tissue culture models of AD, TNFα has been shown to stimulate cell-cycle-mediated neurodegeneration (Bhaskar et al., 2014) and neuronal apoptosis (Blasko et al., 1997). Despite its genetic basis, expression of the A-T disease phenotype is also modulated by the immune system. As example, lipopolysaccharide (LPS)-induced inflammation produces Purkinje cell damage and early stages of neurodegeneration in A-T mouse models (Yang et al., 2014).

We sought to determine which cytokine(s) is/are most responsible for worsening the A-T phenotype and to expand the search for neuronal phenotypic change beyond the cerebellum. We find that, in contrast to cerebellar neurons, the cytokine storm induced by LPS causes little or no damage to frontal cortical neurons. Further, comparing the responses to injections of individual cytokines with those of LPS reveals a complex picture. Much of the LPS-associated damage can be replicated by IL1β alone; yet TNFα, which is also induced by LPS, produces unexpected neuroprotective effects that are particularly evident in *Atm^−/−^* brains. There is also a temporal dimension to the response. We find that wild-type animals recover fully from LPS-injection after 1 month, whereas in *Atm^−/−^* mice evidence of neuronal distress persists. Taken together our results emphasize the delicate balance of pro- and anti-inflammatory actions that are triggered by the various stimulants. Further, the responsiveness of the A-T phenotype to these types of environmental challenges underscores both the role of inflammation in the expression of A-T symptoms and the potential for immune based strategies for disease management.

## Materials and Methods

### *Atm*-deficient mice (*Awb*)

A breeding colony of mice with a targeted disruption of the *Atm^tm1Awb^* gene (Barlow et al., 1996) was obtained from The Jackson Laboratory. Generation of mutants was achieved through the mating of heterozygous *Atm^+/−^* males and *Atm^+/−^* females. The mice were maintained on a 129/SvJ genetic background. Genotyping was performed on extracted tail DNA using PCR techniques described previously (Barlow et al., 1996). All animals were housed at the accredited animal facility of the authors' University. All procedures involving animals were approved by the appropriate local authorities.

### Treatments with LPS or individual cytokines

LPS (*Escherichia coli* serotype 055:B5) was purchased from Sigma-Aldrich (L2880). To mimic the disease progression in human A-T, recombinant human TNFα (#1050) and IL1β (#4128) were chosen and purchased from BioVision. For use, each was dissolved in distilled water and stored at −20°C. Two treatment groups were established. In the first group, adult mice (2–3 months old) of either *Atm* ^+/+^ or *Atm^−/−^* genotype were given daily intraperitoneal injections of LPS (1 mg/kg), TNFα (70 µg/kg), or IL1β (5 µg/kg) diluted in phosphate buffered saline solution, pH 7.4, for a period of 4 d. A control group was treated on the same schedule, but injected with filtered saline only. Mice were killed on the 5^th^ day, 24 h after the last injection. The second group of animals (*Atm* ^+/+^ and *Atm^−/−^*genotype) was injected with LPS or filtered saline following the same schedule but was allowed to recover for 1 month after the last injection before being sacrificed for analysis. Mice in both groups were monitored carefully for signs of sickness or distress during the entire period. Following sacrifice, the brains were dissected and the tissues prepared as described below.

### Tissue preparation and histology

Animals were deeply anesthetized with Avertin (0.02 cc/g body weight) and transcardially perfused with cold PBS for ∼2 minutes. After perfusion, the brains were dissected out and bisected along the midline. One-half of the brain was stored at −80°C until use. The other one-half was fixed in 4% paraformaldehyde at 4°C overnight. After washing with PBS twice, the brain was transferred to a 30% sucrose solution at 4°C overnight for cryoprotection and was embedded in OCT; 10 µm cryostat sections were cut and allowed to air dry on precoated *SuperPlus* glass slides.

### Double-fluorescence immunohistochemistry

Double-fluorescence immunohistochemistry was performed on mouse brain cryosections according to standard methods. Sections were rinsed in PBS, followed by pretreatment at 95-100°C in 0.1M citrate buffer for 8–10 minutes. After the slides had cooled in buffer to room temperature (45–60 minutes), slides were rinsed in PBS. Sections were incubated in 10% goat serum in PBS for 1 h at room temperature to block non-specific binding. All primary antibodies were diluted in PBS containing 0.3% Triton X-100 and 10% goat serum and then were applied to sections and incubated overnight at 4°C. PCNA (mouse, 1:2000), cleaved caspase-3 (rabbit, 1:400), and phospho-p38 (rabbit, 1:100) antisera were purchased from Cell Signaling Technology; Iba-1 (rabbit, 1:250), CD45 (rabbit, 1:70), GFAP (rabbit, 1:2000), γ-H2AX (rabbit, 1:250), HDAC4 (rabbit, 1:100), calbindin (rabbit, 1:500) and MAP2 (chicken, 1:5000) antisera were purchased from Abcam; cyclin A1 (rabbit, 1:100), and cyclin D2 (rabbit, 1:100) antisera were purchased from Santa Cruz Biotechnology. After rinsing in PBS, they were incubated for 2 h at room temperature with secondary antibodies conjugated with fluorescent AlexaFluor dye 647 purchased from Life Technologies and with fluorescent AlexaFluor dye 488 purchased from Jackson ImmunoResearch. Sections were then rinsed in PBS and counter-stained with DAPI for 5 min at room temperature. After rinsing, all sections were mounted with antifading fluorescence media from Vector Laboratories under a glass coverslip. All experiments were conducted in triplicate.

### Cell counting

The method for cell counting in cerebellum was that of Yang et al., (2014). Briefly, five fields were randomly chosen for quantification using a 20× objective on an Olympus or a Leica fluorescent microscope and the percentage of MAP2-labeled Purkinje cells that were also positive for markers of interest were counted. For cell counting in frontal cortex, total MAP2-labeled neurons within layers II–VI that colocalized with markers of interest were counted in a 20× objective field. Calbindin staining was visualized by confocal microscopy (Leica Microsystems).

### Quantitative real-time PCR

Cortex and cerebellum tissues were homogenized in lysis buffer containing 3% β-mercaptoethanol and total RNA was extracted using PureLink RNA mini-kit (Life Technologies) according to the manufacturer’s protocol. Subsequently, 1 µg of total RNA was reverse transcribed into cDNA using PrimeScript II first strand cDNA synthesis kit from Takara Biotechnology. cDNA was diluted 1:10 before use. Real-time PCR was performed using SYBR Premix Ex Taq (Takara Biotechnology) using a 7500 Real-Time PCR System (Applied Biosystems, Life Technologies). ROX II was applied as the reference dye. The sets of primers used are listed as follows; the expression level of *Gapdh* was used for normalization:

*Ym1:* 5′-cagctgggatcttcctacca-3′ (sense), 5′-attctgcattccagcaaagg-3′ (antisense); *Tgfβ1*: 5′- acatgtggaactctaccagaaa-3′ (sense), 5′- ctgccgtacaactccagtga-3′ (antisense); *p21*: 5′- cacaggcaccatgtccaatc-3′ (sense), 5′- acgggaccgaagagacaac-3′ (antisense); *Cd45*: 5′- gggttgttctgtgccttgtt -3′ (sense), 5′- ggatagatgctggcgatgat -3′ (antisense); *Trem2*: 5′- ctggaaccgtcaccatcact -3′ (sense), 5′- aggctagaggtgacccacag -3′ (antisense); *Gapdh*: 5′- ggagaaacctgccaagtatga-3′ (sense), 5′- ggtcctcagtgtagcccaag-3′ (antisense).

### Western blot analysis

Cortex and cerebellum were homogenized separately in RIPA lysis buffer (Millipore) containing protease and phosphatase inhibitors (Roche). Tissue lysates were centrifuged at 15,000 rpm at 4°C for 20 minutes. The levels of protein in the supernatants were determined by Protein Assay solution (Bio-Rad). Protein samples were diluted with 4× loading buffer (composition: 0.02% bromophenol blue, 40% glycerol, 250 mM Tris-HCl, 5% β-mercapethanol) and RIPA lysis buffer to the required concentration and denatured at 95°C for 5 minutes. Protein samples were separated by gel electrophoresis and transferred to nitrocellulose membrane (Bio-Rad). After blocking with TBST containing 5% albumin bovine serum (Sigma) or 5% milk (Bio-Rad), membranes were incubated in p38 (rabbit, 1:1000), phospho-p38 (rabbit, 1:1000), ERK (rabbit, 1:1000), phospho-ERK (rabbit, 1:1000), JNK (rabbit, 1:1000), phospho-JNK (rabbit, 1:1000; Cell Signaling Technology), or actin (goat, 1:5000; Santa Cruz Biotechnology) antisera overnight. After rinsing in TBST, they were incubated in HRP-conjugated secondary antibodies purchased from Cell Signaling Technology or Life Technologies at room temperature for 1 h. Membranes were washed in TBST and protein signals were visualized using ECL substrate reagents (Thermo-scientific). The intensities of the bands were quantified by ImageJ and normalized to actin.

### Statistical analysis

All experiments were conducted with a randomized experimental design. Paired or unpaired Student’s *t* test (Prism, GraphPad software v5) were used to determine the differences in values between different groups and the details are listed in Table 3. *p* < 0.05 was considered to be statistically significant.

## Results

### TNFα and IL1β have opposite effects on cerebellar neurons

**Figure 1:**
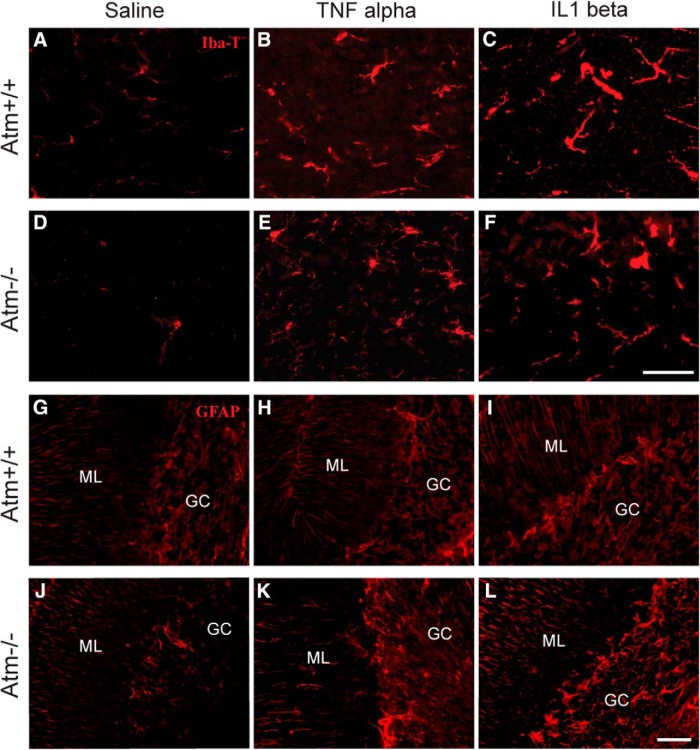
Inflammatory conditions induced by TNFα and IL1β in the cerebellum. TNFα and IL1β triggered microglial activation in both wild-type (***A***–***C***) and *Atm^−/−^* (***D***–***F***). Both cytokines stimulated astrocytic activation in *Atm^−/−^* (***J***–***L***) but not in wild-type (***G***–***I***). GC, Granule cell layer; M, molecular layer. Scale bar, 50 µm.

LPS (intraperitoneal injection) triggers a complex cytokine “storm”; first in the periphery and then in the CNS. Our A-T mouse model cannot replicate the main neurological deficiency (loss of Purkinje cells) in human patients with A-T (Barlow et al., 1996), and so we attempted to use LPS-induced neuronal damage to mimic the neurological defects in human A-T. Although the reactions to systemic injections are complex, we wished to sort out the role(s) of individual proinflammatory cytokines as mediators of the LPS effect. To do this, we injected wild-type and *Atm^−/−^* animals (3 months old) daily with TNFα (70 µg/kg) or IL1β (5 µg/kg) injections for 4 d. As expected, both cytokines triggered the activation of Iba-1-positive microglia in wild-type (Fig. 1*A*–*C*) and *Atm^−/−^*(Fig. 1*D*–*F*) cerebella. When we used GFAP to measure astrocytic activation, however, the cytokines produced evidence of astrogliosis only in *Atm^-/-^* but not in wild-type (Fig. 1*G*–*L*) cerebella. Although our analysis involved both mutant and wild-type animals, only images from mutants are shown.

**Figure 2:**
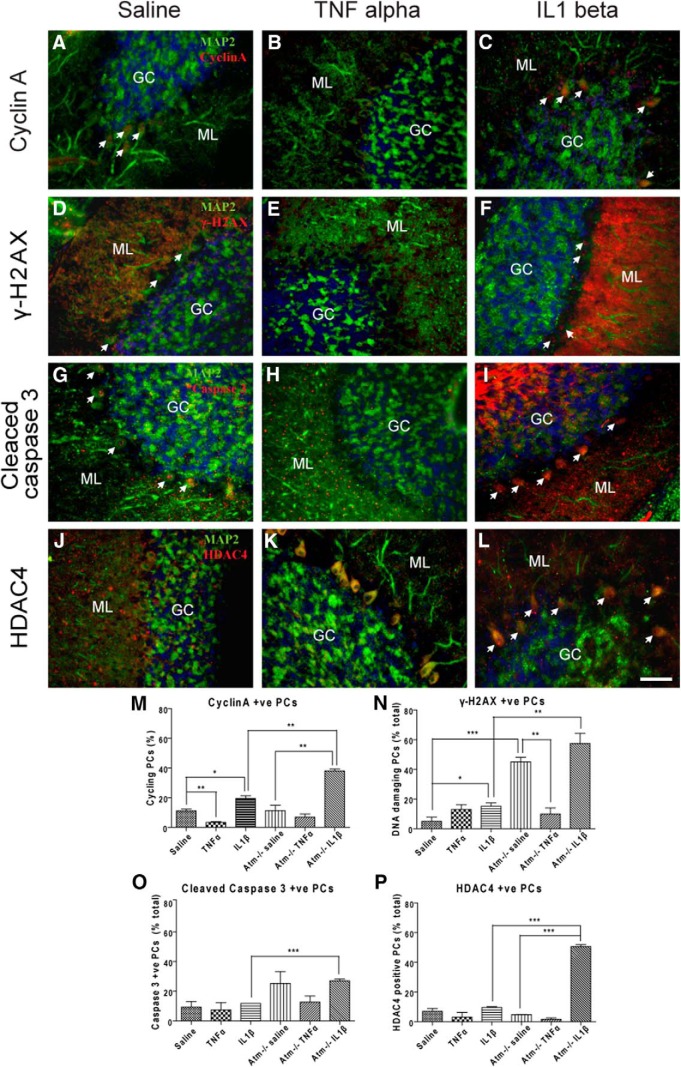
TNFα and IL1β produced opposite responses on cerebellar Purkinje cells in *Atm^+/+^* and *Atm^−/−^*animals. Cyclin A (***A***–***C***), γ-H2AX (***D***, ***E***), cleaved caspase 3 (***G***–***I***), and nuclear HDAC4 (***J***–***L***) were measured after cytokine injection. ATM deficiency induced increase in these markers in Purkinje cells (PCs; ***M***–***P***). TNFα significantly reduced cyclin A in *Atm^+/+^*PCs (***M***) and γ-H2AX in *Atm^−/−^*PCs (***D***, ***E***), whereas IL1β significantly induced cyclin A (***A***, ***C***, ***M***) and HDAC4 nuclear translocation (***J***, ***L***, ***P***). TNFa had no effect or slightly reduced these markers in *Atm^−/−^*PCs. Quantification confirmed these findings (***M***, ***N***, ***P***). Cleaved caspase 3 signals were similar in all treatment groups (***G***–***I***, ***O***). White arrows indicate PCs with respective damage markers. GC, Granule cell layer; ML, molecular layer. Scale bar, 50 µm. *n* = 3 for each group.

We monitored cellular damage in the cerebella of cytokine-injected *Atm^+/+^* and *Atm^−/−^* mice. ATM deficiency led to increased DNA damage in Purkinje cells (Fig. 2*N*). IL1β largely mimicked LPS-induced degenerative changes in *Atm^+/+^* and *Atm^−/−^*Purkinje cells reported by others (Yang et al., 2014), including an increase in cell-cycle events as measured by cyclin A (Fig. 2*A*,*C*,M), increased γ-H2AX staining (Fig. 2*D*,*F*,N), and epigenetic changes as measured by the nuclear translocation of histone deacetylase 4 (HDAC4; Fig. 2*J*,L,P). Interestingly, IL1β drove significantly more neurodegeneration in *Atm^−/−^*than *Atm^+/+^* Purkinje cells (Fig. 2*M*–*P*). Other markers, including cleaved caspase 3 (a cell death marker), were not significantly changed by either TNFα or IL1β within groups with same genotype. Surprisingly, whereas IL1β worsened, TNFα improved the ATM-phenotype in nearly every domain. The effect achieved significance only for DNA damage as measured by cyclin A in *Atm^+/+^* animals (Fig. 2*M*) and γ-H2AX in *Atm^−/−^* animals (Fig. 2*D*,*E*,N); however, all four markers showed a trend toward improvement in *Atm^−/−^*Purkinje cells. The suggestion is that, in the context of our injection protocol, peripheral TNFα administration has neuroprotective qualities rather than the destructive properties expected of a proinflammatory cytokine. As both cytokines would be expected to increase following an LPS injection, a complex response of brain neurons would be predicted.

### IL1β plays a primary role in the neurodegeneration found in *Atm^−/−^*cortex

**Figure 3:**
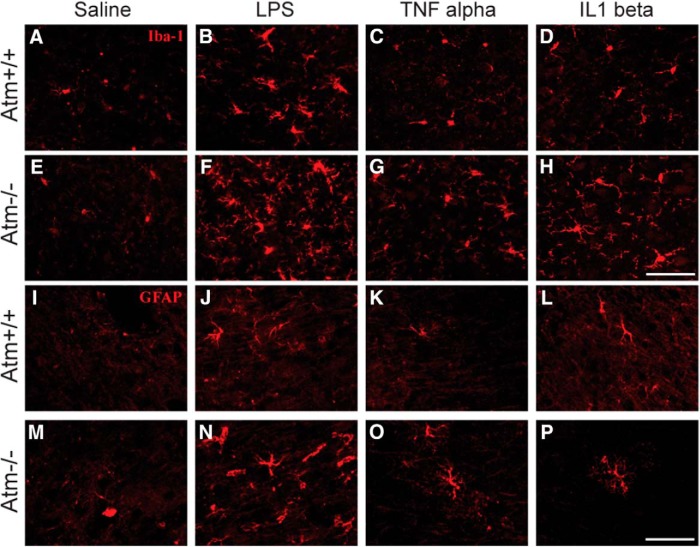
Inflammatory responses induced by LPS, TNFα and IL1β in frontal cortex. In saline-treated animals of both wild-type (***A***) and mutant (***E***) cortex, the microglia appeared in a typical resting phenotype. After LPS treatment (***B***, ***F***), microglia of both genotypes appeared reactive. GFAP, a marker for activated astrocytes, increased in both wild-type (***J***) and *Atm* ^−/−^ mice (***N***) after LPS treatment. Although, TNFα and IL1β produced microglial (***G***, ***H***) and astrocytic (***O***, ***P***) reactions only in *Atm* ^−/−^ frontal cortex. Scale bar, 50µm.

The cerebellum is the brain region that is most dramatically affect in A-T, but neurons and circuits in other brain regions, including cerebral cortex are also affected by the loss of ATM activity (Li et al, 2009, 2012,2013). To explore the response of these other regions to an inflammatory challenge, we repeated the same injection protocols using LPS, TNFα, and IL1β and looked for similarities and differences in the responses of the frontal cortex. Based on the reactive morphology of microglia and astrocytes, LPS administration stimulated a robust inflammatory response in the cortex of both *Atm^+/+^* and *Atm^−/−^*animals (Fig. 3*F*,*N*). TNFα slightly increased reactive microglial morphology in both animal groups (Fig. 3*C*,*G*), whereas IL1β significantly accelerated microglial activation in *Atm^−/−^* but not in *Atm^+/+^* (Fig. 3*D*,*H*) mice. Both TNFα and IL1β slightly changed astrocytic morphology in both wild-type (Fig. 3*K*,*L*) and *Atm^−/−^*(Fig. 3*O*,*P*) mice.

**Figure 4:**
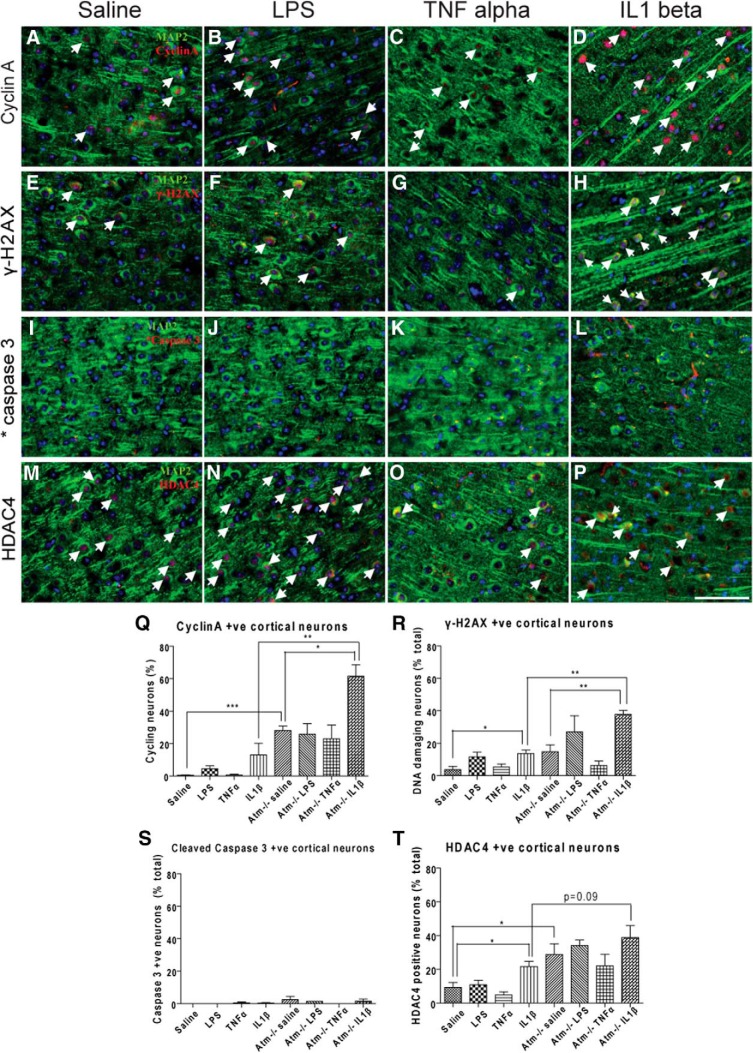
TNFα and IL1β stimulated opposite responses in *Atm^+/+^* and *Atm^−/−^* frontal cortexes. Cyclin A (***A***–***D***), γ-H2AX (***E***–***H***), cleaved caspase 3 (***I***–***L***) and nuclear HDAC4 (***M***–***P***) were measured after LPS or cytokine injection. ATM deficiency significantly increasedγ-H2AX in PCs (***R***). LPS treatment failed to significantly increase the expression of all markers (***B***, ***F***, ***J***, ***N***). TNFα reduced γ-H2AX (***G***), whereas IL1β increased it (***H***). By contrast TNFα had little effect on cyclin A (***C***) or HDAC4 nuclear localization (***O***), whereas IL1β increased both (***D***, ***P***) in *Atm^−/−^* and *Atm^+/+^*frontal cortex (***M***–***P***). Cleaved caspase 3 signals were similar in all treatment groups (***I***–***L***). White arrows indicate neurons with respective damage markers. Scale bar, 50 µm. *n* = 3 for each group.

Similar to cerebellum, ATM deficiency directly induced cellular damage in cortical neurons as shown by cyclin A (Fig. 4*Q*), γ-H2AX (Fig. 4*R*), and HDAC4 (Fig. 4*T*). Although LPS-induced inflammation has effects in both cortex and cerebellum, the cells in two areas respond differently. In frontal cortex, LPS failed to increase evidence for neuronal cell-cycle reentry (Fig. 4*B*,*Q*), DNA damage (Fig. 4*F*,*R*), cell death (Fig. 4*J*,*S*), or nuclear translocation of HDAC4 (Fig. 4*N*,*T*) in both genotypes. This contrasts sharply with the situation in cerebellum where LPS-induced inflammation leads to a substantial increase in Purkinje cell-cycle activity (Yang et al., 2014). Similar to the findings in cerebellum, TNFα failed to accelerate the ATM deficiency-induced damage in both *Atm^+/+^* (Fig. 4*Q*–T) and *Atm^−/−^*neocortex (Fig. 4*C*,*G*,*K*,O); once again the trend for this single cytokine was in the direction of improvement (Fig. 4*R*,*T*). To our surprise, whereas LPS treatment induced few significant changes in frontal cortex, direct injection of IL1β caused multiple deleterious changes. Although the change is not obvious in *Atm^+/+^*neocortex (Fig. 4*Q*,R,T), cyclin A (Fig. 4*D*), and γ-H2AX (Fig. 4*H*) expression significantly increased, whereas nuclear HDAC4 (Fig. 4*P*) expression showed increased trend in IL1β-treated *Atm^−/−^* frontal cortex (Fig. 4*Q*,R,T). There was no evidence for enhanced cell death (cleaved caspase-3 staining) in any of the treatment groups (Fig. 4*J*–*L*,S).

### Neuroprotection is partially determined by MAP kinase signals

**Figure 5:**
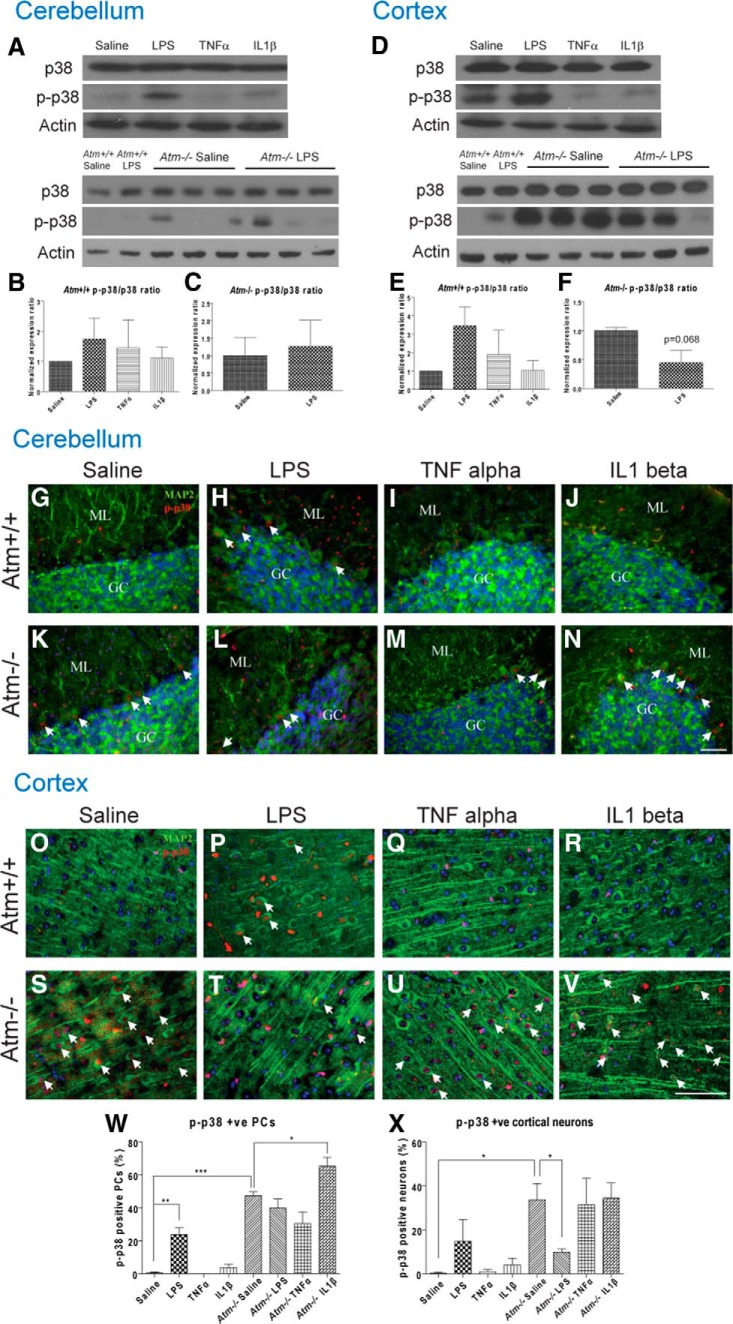
p38 MAP kinase phosphorylation level varied across treatment groups. p38 phosphorylation levels were significantly induced in wild-type cerebellum and cortex following LPS treatment (***A***, ***D***). Quantification showed high variability across each animal (***B***, ***E***). Phospho-p38 immunohistochemistry confirmed the Western results (***G***, ***H***, ***O***, ***P***, ***W***, ***X***). *Atm^−/−^*neurons had increased phospho-p38 levels in both cerebellum and cortex (***G***, ***K***, ***O***, ***S***); quantification confirmed (***W***, ***X***). LPS reduced phospho-p38 levels in *Atm^−/−^* cortex as shown by Western blot (***D***, ***F***) and immunohistochemistry (***S***, ***T***, ***X***) but had no effects on *Atm^−/−^* cerebellum (***A***, ***C***, ***K***, ***L***, ***W***). IL1β induced p38 phosphorylation in *Atm^−/−^* Purkinje cells (***K***, ***N***, ***W***). White arrows indicate neurons with nuclear phospho-p38. GC, Granule cell layer; ML, molecular layer. Scale bar, 50 µm. *n* = 5 for wild-type animals and n = 3 for *Atm^-/-^* animals.

The cytokine signaling pathway has been linked to the activation of the MAP kinases. To explore the mechanism(s) behind the inflammatory responses in cortex and cerebellum we used Western blot analysis and immunohistochemistry to examine both total levels and the phosphorylation status of the various components of this system. The p38 kinase is generally viewed as being elevated during neurodegeneration. Not surprisingly, therefore, on Western blots of wild-type neocortex, the levels of p38 phosphorylation trended higher after LPS treatment (Fig. 5*D*,*E*). Quantification of immunostained tissue (Fig. 5*O*–*R*,X) led to a similar conclusion. Neither of the individual cytokines appeared to have an effect but the high variability across animals may have precluded some results from reaching statistical significance. In cerebellum, we found no detectable change on Western blot (Fig. 5*A*,*B*), but the results were similar when viewed with Purkinje cell immunohistochemistry (Fig. 5*G*–*J*,W).

In the *Atm^−/−^* mutant brain the baseline levels of phospho-p38 were dramatically elevated by the mutation (Fig. 5*A*,*D*). In cortex, LPS paradoxically reduced the phospho-p38 levels in (Fig. 5*F*,S,T,X) even while individual cytokine injections (Fig. 5*U*,V,X) were without obvious effect. IL1β is an effective promoter of cortical damage (Fig. 4), yet after injection no obvious increase in phospho-p38 level was observed in cortical neurons (Fig. 5*X*). In *Atm^−/−^* cerebellum, by contrast, there was no effect of LPS injection (Fig. 5*A*,*C*,*K*,L); however, there was a significant increase in phospho-p38 after IL1β treatment (Fig. 5*N*,*W*). These findings further underscore the complexity of the in vivo situation in the mutant.

**Figure 6:**
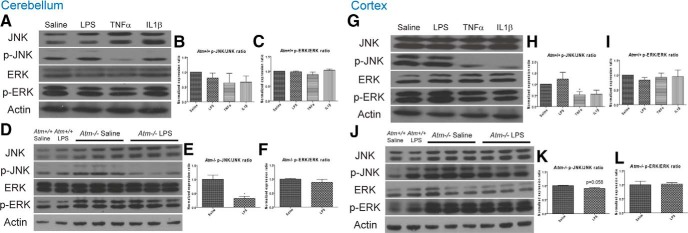
MAP kinase levels in the cerebellum and frontal cortex after different inflammatory challenges. In cerebellum, phosphorylation levels of JNK trended lower after inflammatory stimuli (***A***, ***B***). This effect was enhanced in *Atm* ^−/−^, where LPS significantly reduced the levels of phospho-JNK (***D***, ***E***). In cortex, the reverse situation was found. Phospho-JNK decreased significantly in wild-type after immune challenge (***G***, ***H***), whereas in *Atm* ^−/−^ phospho-JNK only trended lower after LPS. In both regions and both genotypes, the levels of ERK phosphorylation were largely unchanged after an immune stimulus (***C***, ***F***, ***I***, ***L***). *n* = 5 for wild-type animals and *n* = 3 for *Atm^−/−^* animals.

ERK and JNK phosphorylation levels were also investigated. Immunohistochemical findings with the phospho-specific antibodies proved unsatisfactory, and so we restricted our analysis to Western blots. Phosphorylation levels of ERK were similar among the treatment groups in both genotypes and both brain regions (Fig. 6, right, quantification in the histograms). By contrast JNK phosphorylation was sensitive to genotype and brain region, as well as to the nature of the immune challenge. The mutant–wild-type difference could be seen in the response to the LPS injections; a nearly 75% drop in phospho-JNK in mutant cerebellum with virtually no change in wild-type cerebellum (Fig. 6*B* and *E*, respectively). The brain region difference was also apparent after LPS injection. Although there was a 75% drop in phospho-JNK levels in mutant cerebellum, there was only a trend towards lower levels in mutant cortex (Fig. 6*E*,*K*). The results of the TNFα injection led to similar conclusions (Fig. 6*B*,*H*). Finally, we found that the response of wild-type cortex most clearly highlighted the differences among the immune challenges; both TNFα (*p* < 0.05) and IL1β (*p* = 0.069) suppressed phospho-JNK (Fig. 6*G*,H), whereas LPS induced no significant change in the two regions.

### Variation in gene expression during different immune challenges

**Figure 7:**
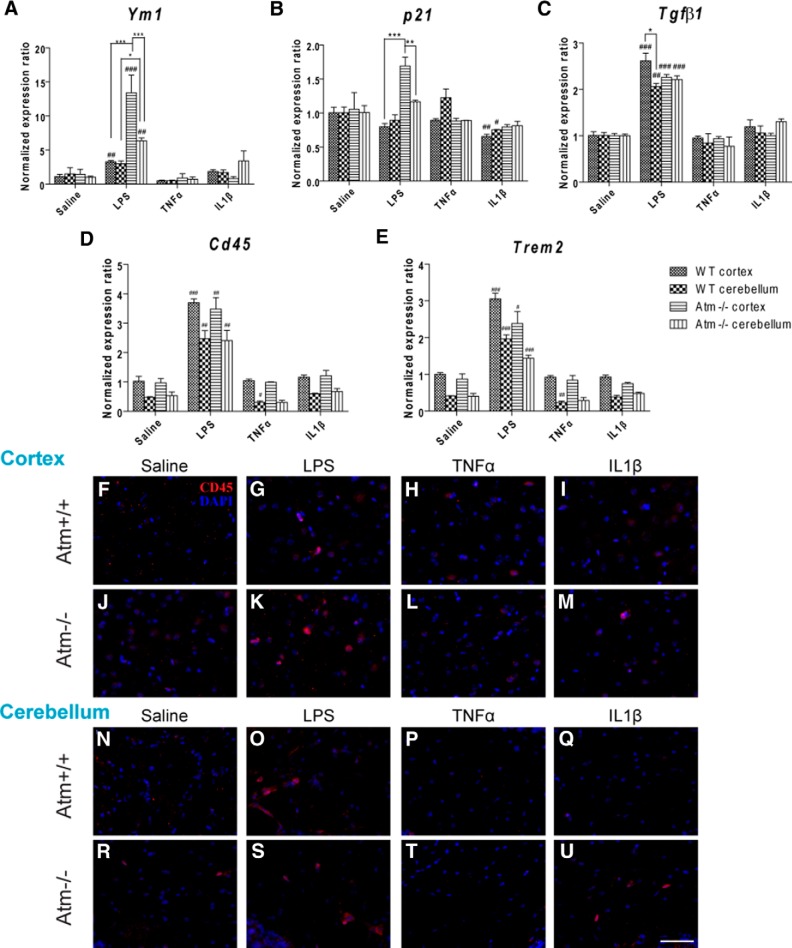
Expression patterns of anti-inflammation associated genes and monocyte infiltration following cytokine stimulation. In wild-type and *Atm^−/−^* mouse cortex, LPS administration significantly triggered the expression of *Ym1* (***A***), *p21* (***B***), *Tgfβ1* (***C***), *Cd45* (***D***), and *Trem2* (***E***); with the exception of a modest change in *p21* expression in wild-type, the individual cytokines were without effect. TNFα specifically suppressed *Cd45* (***D***) and *Trem2* (***E***) in cerebellum of both genotypes. Similar patterns were observed by CD45 protein expression (***F***–***U***). *p < 0.05; **p < 0.01; ***p < 0.001 between cortex and cerebellum in the same treatment group; #*p* < 0.05; ##*p* < 0.01; ###*p* < 0.001 between the values of LPS/cytokine treated and the respective saline-treated cortex or cerebellum groups. Scale bar, 50 µm. *n* = 3 for each group.

The highly variable nature of these responses led us to ask whether a compensatory response of the network of anti-inflammatory genes might be involved. We focused on this hypothesis because we reasoned that if cells were delicately balanced between a pro- and anti-inflammatory phenotype, one would predict exactly the type of variability that we observe. To explore this possibility we used q-PCR to examine the expression of three genes associated with the anti-inflammatory state (also known as M2); *Ym1*, *p21*, and *Tgfβ1* (Fig. 7*A*–*C*). Given the sensitivity of the system to *Atm* genotype, we were surprised to find that the levels of all three genes were unchanged in the untreated mutant brains. Yet when we compared the expression responses of the three to the various immune challenges, we found a broadly similar pattern. LPS induced a significant response in all three genes; the single cytokines were far less effective. *Ym1* and *p21* were far more sensitive in *Atm^−/−^* than in wild-type brain and cortex was more responsive to LPS than cerebellum. *Tgfβ1* was sensitive in both genotypes and both regions (albeit with an enhanced response in wild-type cortex). To discriminate between the contribution of microglia and circulating monocytes to M2 phenotype, gene and protein expression of *Cd45* (a peripheral monocyte marker) was investigated by q-PCR and immunohistochemistry. At the same time, expression of the monocyte specific anti-inflammatory gene *Trem2* (triggering receptor expressed on myeloid cells 2) was also investigated. Although expression of the two genes was lower in cerebellum than cortex, the levels were unchanged in the untreated mutant brains (Fig. 7*D*–*F*). Significantly, LPS induced the infiltration of circulating monocytes in both brain regions of both genotypes as revealed by increased CD45 gene (Fig. 7*D*) and protein (Fig. 7*G*,*K*,O,S) expression. Similar responses were observed for *Trem2* (Fig. 7*E*). No single cytokine induced infiltration of peripheral monocytes while TNFα reduced gene (*Cd45* and *Trem2*) and protein (CD45) expression (Fig. 7*D*,*E*,P,T) in cerebellum but not in cortex of both genotypes.

Together, these data indicate that the upregulation of anti-inflammatory genes during a cytokine challenge occurs, perhaps as a protective reaction against the inflammatory environment. This counteracts some, but not all, of the destructive effects of the proinflammatory response; individual variation in the balance between the two leads to a wide range of responses; from neutral to strongly negative.

### Lack of cellular recovery in *Atm^−/−^* mice after an inflammatory challenge

The anti-inflammatory (M2) response is most typically observed after the front of the cytokine storm has passed. This would suggest that the mice might be engaged in a response that would lead to a reversal of the damage induced by the 4 d LPS challenge. Thus, we repeated the LPS injections described above but allowed the animals to recover for 1 month after the injections.

**Figure 8:**
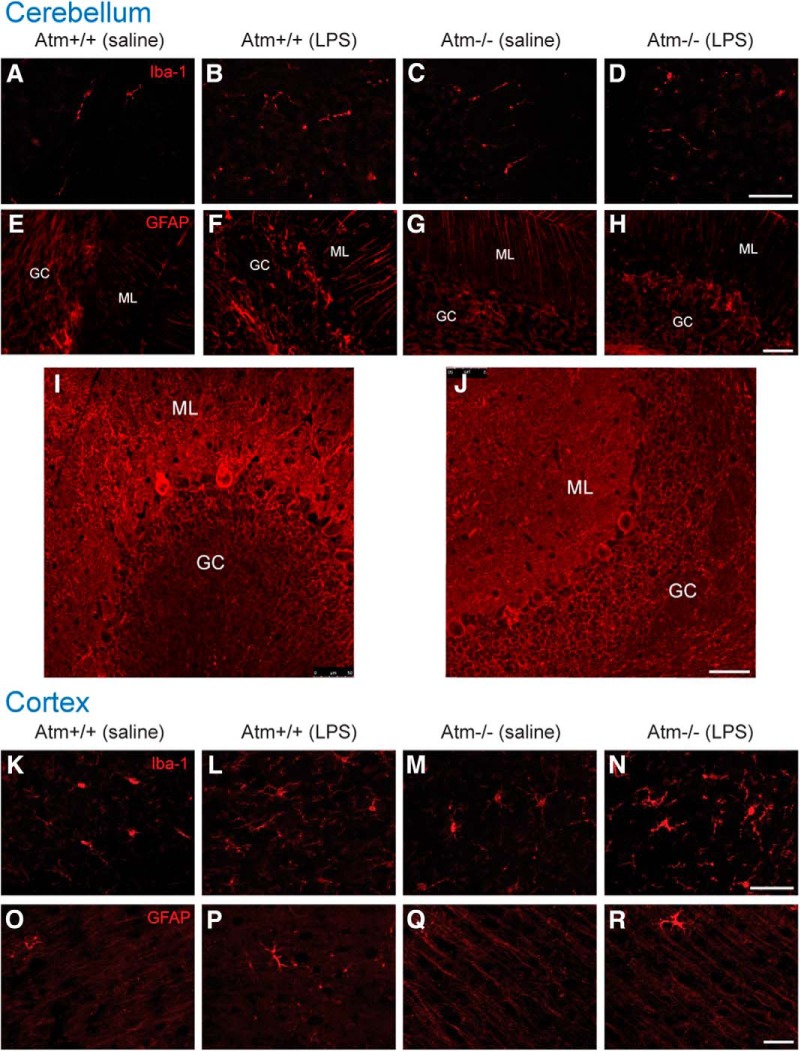
Persistent effects of an immune challenge in *Atm* ^−/−^, but not wild-type mice. In both wild-type and *Atm^−/−^* cerebellum, microglia (***A***–***D***) and astrocytes (***E***–***H***) had returned to resting morphologies after a 1 month recovery. Despite this, calbindin staining revealed that in LPS challenged *Atm^−/−^*mice (***J***) the Purkinje cells remained dystrophic compared with Purkinje cells in untreated *Atm^−/−^*animals (***I***). In cortex, Iba-1 staining revealed the persistence of activated microglia in LPS-treated mutant (*Atm* ^−/−^*)* animals but not in wild-type (***K***–***N***). Astrocyte morphologies returned to resting states in all animal groups (***O***–***R***). GC, Granule cell layer; ML, molecular layer. Scale bar, 50 µm. *n* = 3 for each group.

Iba-1 and GFAP immunohistochemistry showed that astrocyte morphologies returned to normal not only in wild-type, but also in *Atm^−/−^*after the 1 month recovery (Fig. 8*E*–*H*,O–R). The microglial recovery was incomplete in the mutant, however. Whereas in *Atm^−/−^* cerebellum Iba1 cells returned to their resting state (Fig. 8*H*), in *Atm^−/−^*cortex, Iba1-positive cells with an activated morphology persisted even after the 1 month recovery period (Fig. 8*N*). In keeping with this incomplete recovery of the immune response cells, the neurons of the *Atm^−/−^*brain also showed lingering deficits one month after the LPS injections were stopped (Tables 1 and 2). Most prominent among these was the number of cyclin A-positive neurons; these remained significantly higher in LPS-treated *Atm^−/−^*mice (*p* < 0.01) compared with saline-injected mutants. The incomplete recovery of the mutants could also be seen in the appearance of the *Atm^−/−^*Purkinje cell dendritic morphology. Calbindin immunostaining showed continuing structural deficits even 1 month following the LPS challenge (Fig. 8*J*, compare with I, untreated *Atm^−/−^* cerebellum.

**Table 1:** Cerebellar Purkinje neuron labeling after 1 month recovery

	Wild-type (saline)	Wild-type (LPS)	*Atm^−/−^* (saline)	*Atm^−/−^* (LPS)
Cyclin A	3.75 ± 0.35	4.27 ± 1.08	11.83 ± 2.50	**25.2 ± 2.73** **
Cyclin D1	0.33 ± 0.33	0.88 ± 0.06	1.66 ± 0.87	2.42 ± 1.53
γ-H2AX	11.5 ± 2.27	6.21 ± 3.32	45.9 ± 11.61	49.6 ± 14.0
Cleaved caspase 3	8.68 ± 2.16	10.2 ± 3.47	15.2 ± 0.78	14.4 ± 0.31
HDAC4	5.76 ± 2.94	1.76 ± 1.36	11.8 ± 5.32	4.63 ± 1.89

** Plus bold font = *p* < 0.05 compared with the *Atm^−/−^*group injected with saline.

**Table 2:** Cortical neurons labeling after 1 month recovery

	Wild-type (saline)	Wild-type (LPS)	*Atm^−/−^* (saline)	*Atm^−/−^* (LPS)
Cyclin A	1.98 ± 0.96	1.48 ± 0.24	26.58 ± 2.30	38.0 ± 3.31*
Cyclin D1	1.33 ± 0.97	1.48 ± 0.77	3.59 ± 1.09	6.09 ± 3.10
γ-H2AX	2.58 ± 1.74	4.08 ± 2.34	6.53 ± 2.56	8.02 ± 0.95
Cleaved caspase 3	0	0	0	0
HDAC4	4.40 ± 2.27	7.13 ± 2.55	14.6 ± 0.36	22.2 ± 15.3

* Plus bold font = *p* < 0.05 compared with the *Atm^−/−^*group injected with saline.

**Table 3:** Statistical table

	Data structure	Type of test	Power
a (Fig. 2M)	Normally distributed	Unpaired *t* test	1.00
b (Fig. 2M)	Normally distributed	Unpaired *t* test	0.96
c (Fig. 2M)	Normally distributed	Unpaired *t* test	1.00
d (Fig. 2M)	Normally distributed	Unpaired *t* test	0.80
e (Fig. 2N)	Normally distributed	Unpaired *t* test	0.80
f (Fig. 2N)	Normally distributed	Unpaired *t* test	1.00
g (Fig. 2N)	Normally distributed	Unpaired *t* test	1.00
h (Fig. 2N)	Normally distributed	Unpaired *t* test	1.00
i (Fig. 2O)	Normally distributed	Unpaired *t* test	1.00
j (Fig. 2O)	Normally distributed	Unpaired *t* test	0.29
k (Fig. 2P)	Normally distributed	Unpaired *t* test	1.00
l (Fig. 2P)	Normally distributed	Unpaired *t* test	1.00
m (Fig. 4Q)	Normally distributed	Unpaired *t* test	0.99
n (Fig. 4Q)	Normally distributed	Unpaired *t* test	1.00
o (Fig. 4Q)	Normally distributed	Unpaired *t* test	1.00
p (Fig. 4R)	Normally distributed	Unpaired *t* test	0.93
q (Fig. 4R)	Normally distributed	Unpaired *t* test	1.00
r (Fig. 4R)	Normally distributed	Unpaired *t* test	1.00
s (Fig. 4T)	Normally distributed	Unpaired *t* test	0.83
t (Fig. 4T)	Normally distributed	Unpaired *t* test	0.78
u (Fig. 4T)	Normally distributed	Unpaired *t* test	0.60
v (Fig. 5E)	Normally distributed	Paired *t* test	0.27
w (Fig. 5F)	Normally distributed	Unpaired *t* test	0.70
x (Fig. 5W)	Normally distributed	Unpaired *t* test	1.00
y (Fig. 5W)	Normally distributed	Unpaired *t* test	1.00
z (Fig. 5W)	Normally distributed	Unpaired *t* test	0.86
aa (Fig. 5X)	Normally distributed	Unpaired *t* test	0.31
bb (Fig. 5X)	Normally distributed	Unpaired *t* test	0.99
cc (Fig. 5X)	Normally distributed	Unpaired *t* test	0.88
dd (Fig. 6B)	Normally distributed	Paired *t* test	0.21
ee(Fig. 6E)	Normally distributed	Unpaired *t* test	0.98
ff (Fig. 6H)	Normally distributed	Paired *t* test	0.93
gg (Fig. 6H)	Normally distributed	Paired *t* test	0.69
hh (Fig. 6K)	Normally distributed	Paired *t* test	0.70
ii (Fig. 7A)	Normally distributed	Unpaired *t* test	1.00
jj (Fig. 7A)	Normally distributed	Unpaired *t* test	1.00
kk (Fig. 7A)	Normally distributed	Unpaired *t* test	1.00
ll (Fig. 7A)	Normally distributed	Unpaired *t* test	1.00
mm (Fig. 7A)	Normally distributed	Unpaired *t* test	1.00
nn (Fig. 7B)	Normally distributed	Unpaired *t* test	1.00
oo (Fig. 7B)	Normally distributed	Unpaired *t* test	1.00
pp (Fig. 7B)	Normally distributed	Unpaired *t* test	0.98
qq (Fig. 7B)	Normally distributed	Unpaired *t* test	0.87
rr (Fig. 7C)	Normally distributed	Unpaired *t* test	1.00
ss (Fig. 7C)	Normally distributed	Unpaired *t* test	0.87
tt (Fig. 7C)	Normally distributed	Unpaired *t* test	1.00
uu (Fig. 7C)	Normally distributed	Unpaired *t* test	1.00
vv (Fig. 7C)	Normally distributed	Unpaired *t* test	1.00
ww (Fig. 7D)	Normally distributed	Unpaired *t* test	1.00
xx (Fig. 7D)	Normally distributed	Unpaired *t* test	1.00
yy (Fig. 7D)	Normally distributed	Unpaired *t* test	1.00
zz (Fig. 7D)	Normally distributed	Unpaired *t* test	1.00
aaa (Fig. 7D)	Normally distributed	Unpaired *t* test	0.89
bbb (Fig. 7E)	Normally distributed	Unpaired *t* test	1.00
ccc (Fig. 7E)	Normally distributed	Unpaired *t* test	1.00
ddd (Fig. 7E)	Normally distributed	Unpaired *t* test	1.00
eee (Fig. 7E)	Normally distributed	Unpaired *t* test	1.00
fff (Fig. 7E)	Normally distributed	Unpaired *t* test	1.00
ggg (Table 1)	Normally distributed	Unpaired *t* test	0.95
hhh (Table 2)	Normally distributed	Unpaired *t* test	0.81

## Discussion

A-T is a relatively rare autosomal recessive disorder (approximately 1 of 100,000 persons; Swift et al., 1986; Gatti 1995) that causes symptoms across a wide range of organ systems (Lavin and Shiloh 1997; Chun and Gatti 2004; Biton et al., 2008). A-T patients experience a postnatal cerebellar atrophy (Kamiya et al., 2001; Alterman et al., 2007; Bottini et al., 2012; Verhagen et al., 2012) predominantly due to the thinning of the molecular and granular layer (Ciemins and Horowitz, 2000; Verhagen et al., 2012). Heterotopic Purkinje neurons are also found in most cases (Ciemins and Horowitz, 2000; Verhagen et al., 2012) as is an abnormal density of cerebellar stellate cells within the molecular layer (Ciemins and Horowitz, 2000). White matter abnormalities are also found (Kamiya et al., 2001). In the aggregate, these changes in brain cell biology are believed to be the most proximal cause of the neurological symptoms (Perreault et al. 2012).

### Immune system abnormalities in A-T

Due to the lack of DNA repair during V(D)J recombination, abnormalities in the immune system are to be expected in A-T patients. There is general reduction in the numbers of B and T lymphocytes and a gradual decrease in serum immunoglobulin (Ig) levels (Nowak-Wegrzyn et al., 2004; Perreault et al. 2012); but the immune system, while compromised, is not totally disabled. Human studies have shown that the levels of proinflammatory cytokines, such as interleukin 8, are actually elevated in A-T patients (McGrath-Morrow et al., 2010). Similarly, microglial cells and astrocytes with an activated morphology have been reported in A-T cerebellum and cerebral cortex (Liu et al., 2005; Bottini et al., 2012). In the ATM-deficient mouse model, cerebellar astrocytes lose their ability to secrete neurotrophic factors and interact with vascular system (Meshulam et al., 2012).

### The ATM-deficient CNS responds to an immune challenge in a region-specific manner

LPS induces glial activation in both cerebellum (Yang et al., 2014) and frontal cortex (Fig. 3), yet we find that LPS fails to induce the typical indices of cellular damage in the neocortex (Fig. 4). The markers we used cover a wide range of functions including ectopic neuronal cell cycling, caspase-3 activation, DNA damage, HDAC4 nuclear translocation, and the response of the MAP kinase pathways. Although it is perhaps not surprising that differences exist among different brain regions, the mechanisms behind the differences remain almost completely unknown and could well hold important clues to the clinical progression of A-T.

### No single cytokine reproduces the entire spectrum of immune responses

The LPS response is complex, but almost surely involves the proinflammatory cytokines IL1β and TNFα. By injecting each of these two candidate cytokines separately, we sought to highlight their individual contributions to the response. Both cytokines, administered peripherally, led to a mild but apparent microglial and astrocytic activation in cerebellum and cortex of both wild-type and *Atm^−/−^* mice (Fig. 1). But although the microglial and astrocytic responses were similar, the two cytokines worked in opposite ways with respect to the neuronal response. TNFα partially blocks cell cycle activity as well as DNA damage in *Atm^−/−^* neurons, whereas IL1β mimicked LPS in its ability to stimulate neuronal cell cycling, DNA damage and HDAC4 nuclear localization in both cortex and cerebellum. Although IL1β exaggerated cellular damage in *Atm^−/−^* neurons, no obvious neuronal death was observed. This reaction is similar to that observed in the mouse models of Alzheimer's disease where damage (cell-cycle events and DNA damage) begins in early stages (Yang et al., 2006) even though neuronal loss does not appear until later if at all. Also, as in the Alzheimer's mouse models (Dodart et al., 2002) the neuronal damage is mostly reversible (Fig. 8) and this suggests that persistent neuroinflammation is required to drive significant neuronal degeneration and death.

Though typically characterized as proinflammatory cytokines, both TNFα and IL1β have been shown to protect neurons against damage in certain situations. TNFα offers neuroprotection as reflected in the levels of calbindin and superoxide scavenger (Watters and O'Connor, 2011), the up-regulation of potassium channels to block NMDAR-related excitotoxicity (Carlson et al., 1999; Dolga et al., 2008), the regulation of NFκB-related neuroprotection (Taoufik et al., 2011) and the reduction of AD pathology by suppressing amyloid induced Cdk5 activity and tau phosphorylation (Orellana et al., 2007). The evidence for a neuroprotective role for IL1β is less extensive, but it is reported to block NMDA-induced excitotoxicity (Carlson et al., 1999) and to stimulate neurotrophin expression (Song et al., 2013). In our system, the data point to a neuroprotective affect of TNFα and a largely negative impact of IL1β.

We believe that TNFα supplement in A-T animals with neuroinflammation can partially reverse the damage as LPS disrupts blood brain barrier permeability to peripheral factors (Xaio et al., 2001; Gaillard et al., 2003; Pan et al., 2008) and TNFα has been reported to suppress the permeability in rodents (Saija et al., 1995). However, as systemic administration of LPS usually leads to TNFα production in brain (Qin et al., 2007; Xing et al., 2011), more studies are required to define whether TNFα is beneficial to animals that are vulnerable to inflammation.

### Loss of ATM activity affects MAPK signaling and gene expression profile

The cytokine mediated response in the CNS involves the activation of upstream MAP kinase (MAPK) and Akt pathways. These kinase cascades have been shown to play important roles in apoptosis, tumor transformation, and inflammatory or oxidative stress responses in multiple cell types (Hagemann and Blank, 2001). Previous studies have shown a close relationship between ATM activity and activation of MAPKs in cell cycle arrest, senescence, migration and apoptosis in cancer cells (Reinhardt et al., 2007; Ravi et al., 2008; Golding et al., 2009; Wang et al., 2009; Yang et al., 2011b; Yu et al., 2013; Dixit et al., 2014; Hsieh et al., 2014). As ATM has been shown to directly phosphorylate Akt and promote cell survival (Viniegra et al., 2005; Halaby et al., 2008), ATM inhibition is widely used as an anti-tumor strategy in several types of cancer (Golding et al., 2009; Li and Yang 2010). Normally, ATM activated MAPK signaling pathways control cell-cycle checkpoint for DNA repair and promote survival in normal or A-T cells (Panta et al., 2004; Kani et al., 2007; Kim et al., 2007; Barascu et al., 2012) while the ATM-Akt pathway maintains homeostasis and survival by regulating glucose transporter 4 translocation in response to insulin (Halaby et al., 2008), reducing radiosensitivity in response to insulin (Viniegra et al., 2005), nuclear translocation of regulatory precursor microRNAs in response to DNA damage (Wan et al., 2013) and directly activating ERK signaling for DNA repair (Khalil et al., 2011). Interestingly, Akt has been demonstrated to indirectly phosphorylate ATM through an AMP-activated protein kinase ARK5 as the positive feedback mechanism to further promote cell survival (Suzuki et al., 2003). These relationships have led to the proposal that cytoplasmic ATM is a potential therapeutic target for diabetes, cancer, and neuronal degeneration (Yang et al., 2011a).

Among the three MAPK families, p38 MAPK is largely involved with ATM deficiency induced symptoms in the CNS as ATM has been shown to regulate p38 MAPK activity (Thornton and Rincon, 2009). ATM deficiency leads to the loss of self-renewal and proliferative activity in neural stem cells from the subventricular zone due to overactivation of p38 activity; p38 inhibition can restore these functions (Kim and Wong, 2009, 2012; Kim et al., 2011). More importantly, p38 inhibition also improves the motor deficits in *Atm^−/−^* mice, suggesting an essential role of MAPKs in A-T progression (Kim and Wong, 2012). In our A-T model, the phosphorylated forms of all three MAP kinases are elevated in *Atm^−/−^* cortex and cerebellum, consistent with the previous findings (Stern et al., 2002; Liu et al., 2005; Kim and Wong, 2012). Our data from wild-type cortex, show a trend for LPS to drive up p38 phosphorylation levels (pro-degeneration), whereas TNFα significantly blocked JNK phosphorylation (pro-survival). This variable response to different immune challenges was even more apparent in *Atm^−/−^* brain, thus emphasizing the interaction between *Atm* genotype and inflammatory response. (Figs. 5, 6). As ATM has been shown to indirectly activate p38 MAPK (Thornton and Rincon, 2009), we hypothesize that LPS further suppresses ATM activity and this may lead to disruption of MAPK signaling in *Atm^−/−^*brain. Rather than serve as part of the degenerative process, it seems more likely that these shifts represent the final homeostatic defense of the cell against ensuing cell death. Although the LPS response is no doubt multifaceted (with both protective and destructive components overlapping), it is noteworthy that IL1β, when injected alone, raised rather than lowered phospho-p38 levels in *Atm^−/−^* Purkinje neurons. We propose that this identifies IL1β as one of the main proinflammatory cytokines that leads to the behavioral deficits and chronic degeneration in A-T.

### Interaction of the cytokines

Preconditioning and short-term treatment of LPS have been shown to offer neuroprotection against ischemic hypoxia and brain injuries in rodents (Rosenzweig et al., 2004; Lin et al., 2009; Chen et al., 2012). We propose that this largely unexpected response is mimicked in our system and may be explained by the enhanced sensitivity of anti-inflammatory genes such as *Ym1*, *p21*, and *Tgfβ1* to a deficiency of ATM kinase. In the absence of an immune stimulus, the levels of all three M2-related genes are nearly identical in wild-type and *Atm^−/−^* brain; in both cortex and cerebellum. After LPS injection, however, the levels of *Ym1*, *p21*, and *Tgfβ1* all increased dramatically in mutant cerebellum and even more so in cortex (Fig. 7). Previous findings have shown that LPS could stimulate brain infiltration of peripheral neutrophil and monocyte (Ji et al., 2008; Zhou et al., 2009; Jeong et al., 2013) and so peripheral immune cells would contribute to the M2 phenotype. As demonstrated by *Cd45* gene and protein and *Trem2* gene levels, LPS potentially induces monocyte infiltration and these M2 monocytes could help repair the inflammation induced damage. This dramatic response does not appear to be mediated by either IL1β or TNFα alone as the individual cytokines produced little change in expression in any of the three genes. However, TNFα could reduce monocyte infiltration in cerebellum (Fig. 7). This suggests that TNFα exerts its neuroprotective function by maintaining BBB permeability (Saija et al., 1995) and suppressing infiltration of M1 monocytes in neurodegenerative diseases. The contribution of ATM deficiency to the LPS response is emphasized by the fact that in the wild-type brain, LPS induced at best a modest increase in the mRNA for *Ym1* and *Tgfβ1*, whereas p21 message levels trended lower. Together with the Western blot studies, we suggest that LPS is working in both “good” and “bad” directions in the brain and that the ultimate effect is determined by both genotype and brain region.

### Chronic inflammation affects long-term cortical and cerebellar functions

Although the detailed mechanism remains to be worked out, the persistent deficits seen in *Atm^−/−^* mice one month after an LPS immune challenge raise questions with important clinical significance. The 4 week recovery period allowed wild-type brains ample time to restore all markers, both cellular and biochemical, to their resting state. Significantly however, in the *Atm^−/−^* brain, the expression of some markers persisted well into this long recovery period. The levels of cleaved caspase-3, γ-H2AX, the enhanced nuclear localization of HDAC4 and the strong astrocytic response all return to near baseline. But the microglial response, though reduced, did not completely return to the pre-LPS levels (compare Figs. 3 and 8) and the expression of some cell cycle markers, notably cyclin A persisted. More significantly, the atrophy of the Purkinje cell dendritic tree, as seen in calbindin immunostaining, never fully recovered, suggesting the persistence of long-term structural damage to the cellular elements of the nervous system. The reason for this vulnerability of the *Atm^−/−^* neurons is unknown. Perhaps the inflammatory process induces large numbers of DNA breaks that would be repaired more slowly in the absence of ATM. Or perhaps the loss of the neuron-specific and cytoplasmic functions of ATM makes neurons particularly slow to recover from the LPS injections. Quite possibly both these and other *Atm^−/−^*phenotypes contribute to the problem. Viewed from the perspective of human A-T, the strong suggestion is that control of inflammation, even in patients with already compromised T- and B-cells, should be of significant clinical benefit.

For each statistical test run in the study, the data structure, statistical test, and power are listed.

## Synthesis

The decision was a result of the Reviewing Editor Orly Reiner and the peer reviewers coming together and discussing their recommendations until a consensus was reached. A fact-based synthesis statement explaining their decision and outlining what is needed to prepare a revision is listed below. The following reviewers agreed to reveal their identity: Roberto Giovannoni, Da-Qing Yang

The manuscript describes the potential effects of inflammatory response on neurological phenotype progression with regional differences in the CNS response to lesion in ataxia telangiectasia model. These findings provide advances in the field of the understanding how the differential response of injured cells can explain the disease progression and potentially lead to new therapeutic target identification.

The authors satisfactorily addressed all the points/concerns raised by the reviewers.
